# Immunological Activity Difference between Native Calreticulin Monomers and Oligomers

**DOI:** 10.1371/journal.pone.0105502

**Published:** 2014-08-29

**Authors:** Mi-chun He, Jun Wang, Jian Wu, Fang-yuan Gong, Chao Hong, Yun Xia, Li-juan Zhang, Wan-rong Bao, Xiao-Ming Gao

**Affiliations:** 1 Institutes of Biology and Medical Sciences, Soochow University, Suzhou, Jiangsu Province, China; 2 Internal Medicine Center of Rheumatoid Diseases, the First Hospital of Soochow University, Suzhou, Jiangsu Province, China; Institut National de la Santé et de la Recherche Médicale U 872, France

## Abstract

We have recently demonstrated that the greatly increased immunological activities of recombinant murine calreticulin (rCRT) are largely attributed to its self-oligomerization. Although native CRT (nCRT) can also oligomerize under stress conditions in vitro, whether this phenomenon could occur inside cells and the immunological activity difference between nCRT monomers and oligomers remained unclear. In this study, we illustrated the formation of CRT oligomers in tranfectant cells under “heat & low pH” (42°C/pH 6.5) condition. The mixture of nCRT oligomers and monomers (OnCRT) was obtained after 3 hr treatment of murine monomeric nCRT (MnCRT) under similar condition (42°C/pH 5.0) in vitro. The OnCRT thus obtained was better recognized by 2 monoclonal Abs from mice that had been immunized with oligomeric rCRT. Unlike MnCRT, OnCRT was able to elicit CRT-specific IgG production in mice. OnCRT also stimulated bone-marrow derived dendritic cells (BMDCs) to secrete significantly higher levels of TNF-α, IL-6 and IL-12p40 than did MnCRT in vitro. We postulate that oligomerization of soluble CRT may occur under certain pathophysiological conditions (e.g. ultrahyperpyrexia) and the resultant oligomers may exhibit exaggerated immunostimulating activities, thereby affiliating the inflammatory responses in vivo.

## Introduction

Calreticulin (CRT) is a calcium-binding endoplasmic reticulum (ER) residential glycoprotein, which contains a globular N domain, a proline-rich P domain, and a Ca^2+^-binding C-domain [1]–[4]. The acidic C domain is an important mediator of calcium-dependent changes in secondary structure and thermostability [5]. Although a classical ER protein, CRT also appears at the cell surface [6], [7], and in soluble form in body fluids such as the blood and synovial fluid [8], exhibiting various immunological functions. For example, cell surface CRT is involved in antigen processing and presentation [9], the uptake of CRT-expressing cancer cells by dendritic cells and phagocytosis of apoptotic cells [6], T cell adhesion, proliferation and function [10], [11], and thrombospondin 1-mediated lymphocyte migration [12]. Soluble rCRT fragments were also shown to be very potent stimulators against macrophages and B cells [13].

Anti-CRT auto-antibodies have been found in sera of patients with various autoimmune diseases such as systemic lupus erythematosus (SLE), rheumatoid arthritis (RA), Sjögren's syndrome, mixed connective tissue disease [14], [15]. Much progress has been made in explaining how this self antigen might be involved in inflammatory auto-immune reponses, with several potential mechanisms, such as shared epitope-mediated recognition and cross-reactive immune response proposed [16], [17].

It has been illustrated by previous investigators that nCRT has the potency to oligomerize in response to heat shock, low pH, calcium depletion, or other forms of physicochemical stress in vitro [18], [19], and rCRT contains both monomers and oligomers [13], [20]. Important questions with regard to nCRT oligomerization are whether and how it would affect the biological function of soluble nCRT in vivo. In the case of Aβ and other amyloidogenic proteins, their pathological activities lie not in the monomers or the insoluble fibrils but rather in the soluble oligomeric intermediates [21]–[23]. Jeffery et al argued that CRT oligomerization could substantially impact its chaperone activity [24]. Our recent work also showed that rCRT oligomers exhibited much stronger immunological activities than its monomeric counterpart [13].

The present work was set out to characterize the immunological functions of oligomeric nCRT formed under heat shock & low pH conditions. In the bimolecular fluorescence complementation (BiFC) experiments, we demonstrated that the combination of high temperature (42°C) and low pH value (6.5) elicited the oligomerization of intracellular CRT. Next, we purified nCRT from murine livers and subsequently induced the formation of oligomers in solution for functional analysis, and found that oligomeric nCRT exhibited enhanced immune stimulating ability both in vitro and in vivo. These data showed that nCRT oligomerization could trigger or exacerbate immune reactions, and thereby further our understandings on the pathophysiological processes underlying many immune-mediated disorders.

## Materials and Methods

### Ethic statements

The approval for use of animals in research was granted by the Animal Care and Use Committee for Health Sciences of Soochow University (SYSK-(S2012-0062)). All animal experiments were carried out according to the Animal Care Guidelines of Soochow University. The animals were housed under specified-pathogen-free (SPF) conditions that meet international standards; they were regularly checked by the certified veterinarian responsible for health monitoring, animal welfare supervision, experimental protocols and procedure revision. At the time to sacrifice they were euthanized by cervical dislocation.

### Reagents

mAb153.24 and mAb153.9 were provided by Dr. Boquan Jin (Forth Military Medical University, Xia'an, China), they were generated by immunizing mice with rCRT oligomers. mAb153.24 recognizes a linear epitope and mAb153.9 recognizes a conformational epitope within the sequence of rCRT/39–272 [20], the specificity of the Abs was shown in Fig. S1 A&B. APC-anti-CD11c were purchased from Biolegend. LPS and BSA were purchased from Sigma-Aldrich.

### Purification of nCRT

nCRT was purified from mouse livers essentially as described before [25], [26]. Briefly, mice were euthanized by cervical dislocation, mouse livers were gently disaggregated and liver cells were collected via centrifugation at 500 g for 5 min. The cell pellet was lysed in 3 volumes of lysis buffer (1% Triton-X 100, 0.2 mM PMSF in PBS) for 30 min on ice with soft stirring followed by centrifugation at 35,000 g for 30 minutes. The resulting supernatants were sequentially precipitated with 50% and 85% saturated (NH_4_)_2_SO_4_ on ice with centrifugation at 35,000 g for 60 min in between. Afterwards, the final precipitate was dissolved in binding buffer (150 mM NaCl, 20 mM Tris, PH 7.4) and subsequently dialyzed against this buffer. The sample was applied to a DEAE Sephadex A50 column (GE Healthcare, US) which was then sequentially washed with binding buffer and washing buffer (280 mM NaCl, 20 mM Tris, PH 7.4) at 1 ml/min to remove contaminating proteins. The fractions were eluted with a linear salt gradient (280–500 mM NaCl) and characterized by SDS-page and western blotting. The procedures of nCRT preparation were under strict sterile conditions as far as possible, and nCRT samples were tested using LPS ELISA Kit (CEB526Ge, Cloud-Clone Corp.), results showed that LPS levels were below 1 pg/ml.

### Western blotting

The SDS-PAGE and native-PAGE gels were electro-blotted onto PVDF membranes, at a constant current of 250 mA in trans-buffer (50 mM Tris, pH 8.0, containing 0.192 M glycine and 20% methanol), using a Bio-Rad Trans-Blot Cell. The PVDF membranes were incubated for 1 hr at room temperature in blocking buffer (TBS containing 5% nonfat milk), followed by an overnight incubation at 4°C with constant agitation in a 1/1000 dilution of rabbit anti-human CRT polyclonal Abs (Affinity BioReagents, Golden, CO) in blocking buffer. After 3 washes with TBS containing 0.05% Tween 20, PVDF membranes were incubated for 1 hr with HRP-conjugated secondary antibody (Southern Biotechnology Associates Inc., USA) and visualized using the ECL detection system as recommended by the manufacturer (Applygen Technologies Inc., Beijing, China).

### Induction of nCRT oligomerization

For induction of nCRT oligomerization by heat & low pH, purified murine nCRT was dialyzed against PBS of pH 5.0, and then placed in the 42°C water bath for an indicated period of time. Formation of nCRT oligomers was visualized by native-page and western-blot. Throughout this study, the untreated nCRT preparations were referred as monomeric nCRT (MnCRT), while nCRT preparations thus treated were designed as OnCRT, albeit the latter apparently contain oligomeric as well as monomeric nCRT molecules. The endotoxin levels in these MnCRT and OnCRT samples were below 1 pg/ml as determined by a LPS ELISA Kit (CEB526Ge, Cloud-Clone Corp.).

### ELISAs

ELISA plates were coated at 4°C overnight with MnCRT or OnCRT (3 µg/ml) and subsequently incubated with blocking solution (1% BSA in PBS) for 2 hrs at 37°C. The wells were washed five times with PBS containing 0.05% Tween 20 (PBS-T) prior to incubation at 37°C with mAb or sera from immunized mice in serial dilution. After 5 washes with PBS-T, the plates were further incubated with HRP-labeled goat-anti mouse for 1 hr at 37°C. The reaction was developed with 100 ul of o-phenylenediamine (OPD, Sigma) for 15 min and stopped with 50 ul 2 M H_2_SO_4_, Optical density (OD) was measured at 492 nm in an ELISA spectrophotometer (Titertek Multiscan Plus MK II; ICN Flow Laboratories, Irvine, UK).

### Cell culture

Cells were cultured in complete RPMI 1640 or Dulbecco's modified eagle medium (DMEM) with high glucose supplemented with 10% (v/v) FBS (HyClone Laboratories, Logan, UT), 100 U/ml penicillin, 100 mg/ml streptomycin, 2 mM L-glutamine and 5×10^−5^ M 2-ME.

For the generation of mouse bone marrow derived DCs (BMDCs), 3×10^6^ bone marrow cells per well in a 6-well plate were cultured in medium supplemented with IL-4 and GM-CSF (15 ng/ml) (Calbiochem, USA). After 7 days, the cells were typically 90% positive for CD11c as determined by FACS analysis.

BMDCs (1×10^5^ cells/well) or splenocytes (2.5×10^5^ cells/well) were stimulated with MnCRT, MnCRT/pH 5.0, OnCRT, BSA (Sigma, 30 mg/ml), or LPS (Sigma, 300 ng/ml) in RPMI1640 medium in 96-well culture plates at 37°C for 48 hrs or 6 days respectively. The concentration of TNF-α, IL-6, IFN-γ, IL-12p40 or IgG in culture supernatants was determined by commercial ELISA kits (BioLegend) following the manufacturer's instructions.

### Mice and Immunization

C57BL/6 mice (female and 6–8 weeks old) were purchased from the Model Animal Research Center, Nanjing, China. All animals were maintained under SPF conditions and all procedures were conducted according to protocols approved by the Soochow University Institutional Animal Care and Use Committee. Mice were immunized s.c. at the back with 150 ug MnCRT or OnCRT dissolved in 100 ul PBS (3 times and interval of 9 days, 5 mice per group). Sera were collected by tail bleeding, aliquoted and stored at −20°C until use.

### The BiFC experiments

To construct the expression vector for the BiFC assay, cDNA enconding the N domain (VN, aa residues 1–172), C domain (VC, aa residues 155–238), or full length Venus protein (VF, aa residues 1–238) were amplified by PCR from an expression vector kindly provided by Prof. Zhao Yun (The Cyrus Tang Hematology Center of Soochow University). They were then fused with the CRT cDNA by PCR techniques through a linker sequence (Gly4Ser)3, generating encoding sequences for fusion proteins of VN-CRT, VC-CRT and VF-CRT, which were inserted into the Hind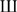/BamHI site of the pcDNA.3.1(+) vector using the primers shown in Table 1.

**Table 1:** Primers for BiFC experiment.

	Forward (5′- 3′)	Reverse (5′- 3′)
VN fragment	GAGCAAGCTTGTCTGGTGAGCAAGGGCGAGGAG	GACCGGGATCCCGCTACTCGATGTTGTGGCGGATC
VC fragment	GAGCAAGCTTGTCATGGACAAGCAGAAGAACGGCATC	GACCGGGATCCCGCTACTTGTACAGCTCGTCCATG
VN fragment for fusion protein	GAGCAAGCTTGTCATGGTGAGCAAGGGCGAGGAG	CCTCCGCCTCCGCTTCCGCCTCCGCCCTCGATGTTGTGGCGGATC
VC fragment for fusion protein	GAGCAAGCTTGTCATGGACAAGCAGAAGAACGGCATC	CCTCCGCCTCCGCTTCCGCCTCCGCCCTTGTACAGCTCGTCCATG
Venus fragment for fusion protein	GAGCAAGCTTGTCATGGTGAGCAAGGGCGAGGAG	CCTCCGCCTCCGCTTCCGCCTCCGCCCTTGTACAGCTCGTCCATG
CRT fragment for fusion protein	GGCGGAGGCGGAAGCGGAGGCGGAGGAAGCGGCGGTGGCGGCAGCGACCCTGCCATCTAT	GACCGGGATCCCGCTACAGCTCATCCTTGG
VN-CRT	GAGCAAGCTTGTCATGGTGAGCAAGGGCGAGGAG	GACCGGGATCCCGCTACAGCTCATCCTTGG
VC-CRT	GAGCAAGCTTGTCATGGACAAGCAGAAGAACGGCATC	GACCGGGATCCCGCTACAGCTCATCCTTGG
VF-CRT	GAGCAAGCTTGTCATGGTGAGCAAGGGCGAGGAG	GACCGGGATCCCGCTACAGCTCATCCTTGG

293T cells, purchased from Institute of Biochemistry and Cell Biology (CAS), cultured in 24-well plates, were transfected with appropriate DNA constructs (250 ng vectors/well) using lipofectamine (5 ul/well, Invitrogen) for transient expression. The cells were harvested 24 hrs post transfection and seeded into 4 different plates, and continued to be cultured for an additional 24 hrs. For the final 3 hrs of incubation, the cells were maintained under either normal (37°C/pH 7.4), or heat shock (42°C/pH 7.4), or low pH (37°C/pH 6.5), or “heat & low pH” (42°C/pH 6.5) conditions. After washing with PBS, the cells were fixed in 1% paraformaldehyde, counterstained with 1 ug/ml DAPI and stored at 4°C until confocal microscope analysis (Nikon system A1).

### Statistical Analysis

All experiments were repeated at least 3 times and the results were expressed as mean ± standard deviation of the mean (SD). Statistical analysis was performed using the Independent-Samples t test between groups using the SPSS 15.0 program (SPSS, Chicago, IL). Differences were considered statistically significant as p<0.05.

## Results

### Intracellular CRT oligomerization induced by heat shock and low pH

**Figure 1:**
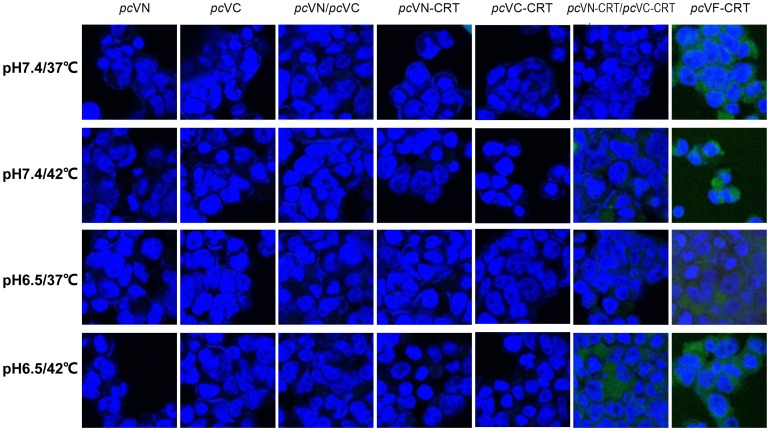
Evidence for intracellular CRT oligomerization. 293T cells were transfected with *pc*VN, or *pc*VC, or *pc*VN-CRT, or *pc*VC-CRT, or *pc*VF-CRT, or a mixture of *pc*VN and *pc*VC or *pc*VN-CRT and *pc*VC-CRT using lipofectamine. After a 48 hr incubation period at 37°C & 5% CO_2_, the cells were cultured for an additional 3 hrs at regular (37°C/pH 7.4, the upper panel), or “heat” (42°C/pH 7.4, the second panel), or “low pH” (37°C/pH 6.5, the third panel), or “heat & low pH” (42°C/pH 6.5, the bottom panel) conditions. The cells were then imaged with confocal microscopy after fixation and staining with DAPI (blue). Green signal denotes the location of VF-CRT protein or VN-CRT/VC-CRT oligomers formed in cells. The results are representatives of three independent experiments.

Previous research showed that purified nCRT monomers could oligomerize in vitro under heat shock or acidic conditions [18], [19]. The BiFC system, which takes the advantage that fluorescence signals can only be observed in cells when N (VN) and C (VC) fragments of the intracellularly expressed Venus protein (VF) are in close proximity through aggregation [27], [28], can be used to examine whether a given protein species (fused with VN and VC fragments of the Venus protein and co-expressed in the cell) could oligomerize inside the cell. Indeed, strong fluorescence signal was readily detected in 293T cells that had been transfected with the expression vector *pc*VF-CRT (encoding for fusion protein of VF-CRT) (Fig. 1). On the other hand, 293T cells transfected with either *pc*VN or *pc*VC (encoding VN and VC fragments, respectively) alone, or co-transfected with both plasmids together, did not produce positive results in parallel experiment, no positive signal was detected even after 3 hrs treatment of “heat shock” (42°C/pH 7.4), or “low pH” (37°C/pH 6.5), or “heat & low pH” (42°C/pH 6.5) conditions (Fig. 1). These results indicated that intracellular aggregation (oligomerization) of the VN and VC fragments could not occur in the cells. We then repeated these experiments using expression vectors encoding fusion proteins VN-CRT and VC-CRT. As illustrated in Fig. 1, cells co-transfected with *pc*VN-CRT and *pc*VC-CRT produced strong fluorescence signals after a “heat & low pH” (42°C/pH 6.5) treatment, while all the other groups did not show obvious fluorescence signals. We therefore conclude that the intracellular aggregation of the VN-CRT and VC-CRT fusion proteins occurred intracellularly under a “heat & low pH” condition, apparently due to oligomerization of their fused CRT tail.

### In vitro induction of nCRT oligomerization

**Figure 2:**
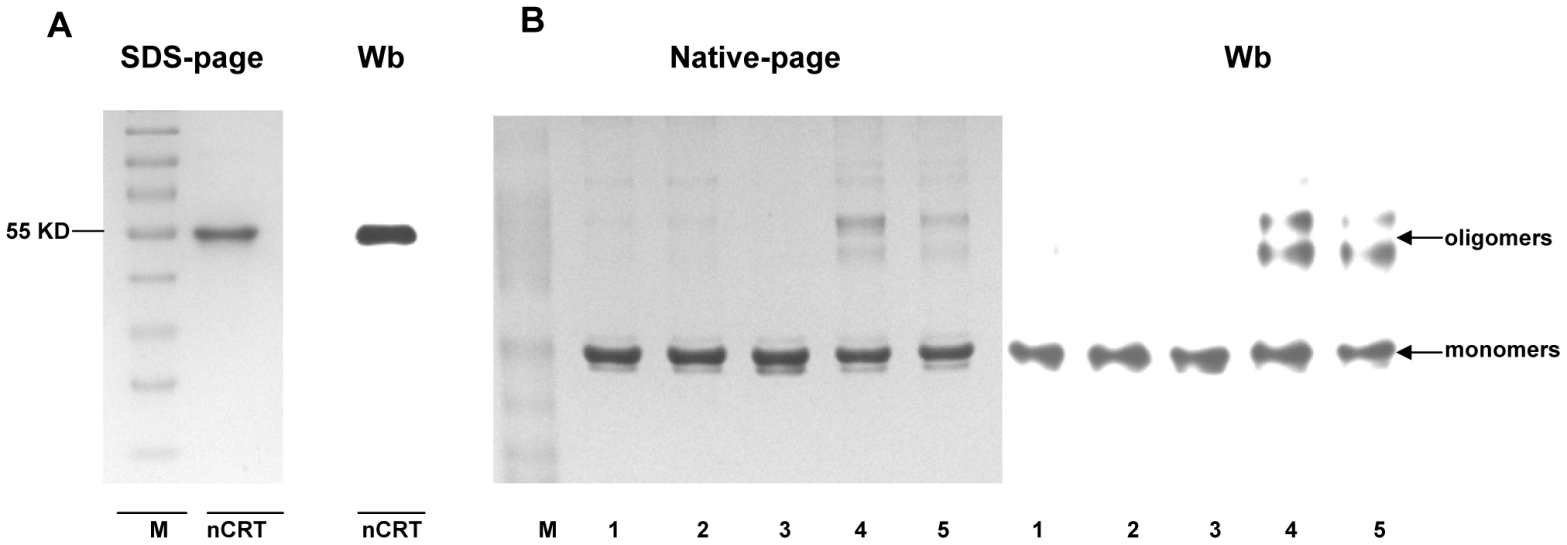
Induction of nCRT oligomerization in vitro. A sample of nCRT purified from murine livers was run on 10% SDS-page gel and analyzed by Western blotting using polyclonal rabbit Abs against CRT (**A**). Samples of nCRT were incubated for 3 hrs under pH 7.4/37°C (Lane 1), or pH 7.4/42°C (Lane 2), or pH 5.0/37°C (Lane 3), or pH 5.0/42°C (Lane 4), or “pH 5.0/42°C” group was maintained for another 3 hrs in conditions of pH 7.4/37°C (Lane 5) prior to native-page gel analysis followed Western blotting (**B**). The results are representatives of three independent experiments.

In order to further characterize the immunological function of nCRT, we purified nCRT from mouse livers by (NH_4_)_2_SO_4_ precipitation combined with ion exchange chromatography. SDS-PAGE and Western-blot assays confirmed that purity of the 55 KD nCRT was more than 90% (Fig. 2A). Native-page study showed that majority of the purified nCRT existed as monomers under physiological conditions (Fig. 2B). When purified nCRT was treated for 3 hrs under “heat & low pH” (42°C/pH 5.0) condition, however, a substantial percentage of the molecules became oligomerized, while treatment conditions such as “heat only” (42°C/pH 7.4) or “low pH only” (37°C/pH 5.0) did not have the same effect (Fig. 2B). Oligomers of the “heat & low pH” group appeared to be stable, which did not return to monomeric form after further incubation at 37°C/pH 7.4 for up to 3 hrs (Fig. 2B). In subsequent experiments, the untreated nCRT preparations were used as monomeric nCRT (MnCRT), while the “heat & low pH”-treated nCRT as oligomeric nCRT (OnCRT).

### Evidence for antigenicity alteration in nCRT following oligomerization

**Figure 3:**
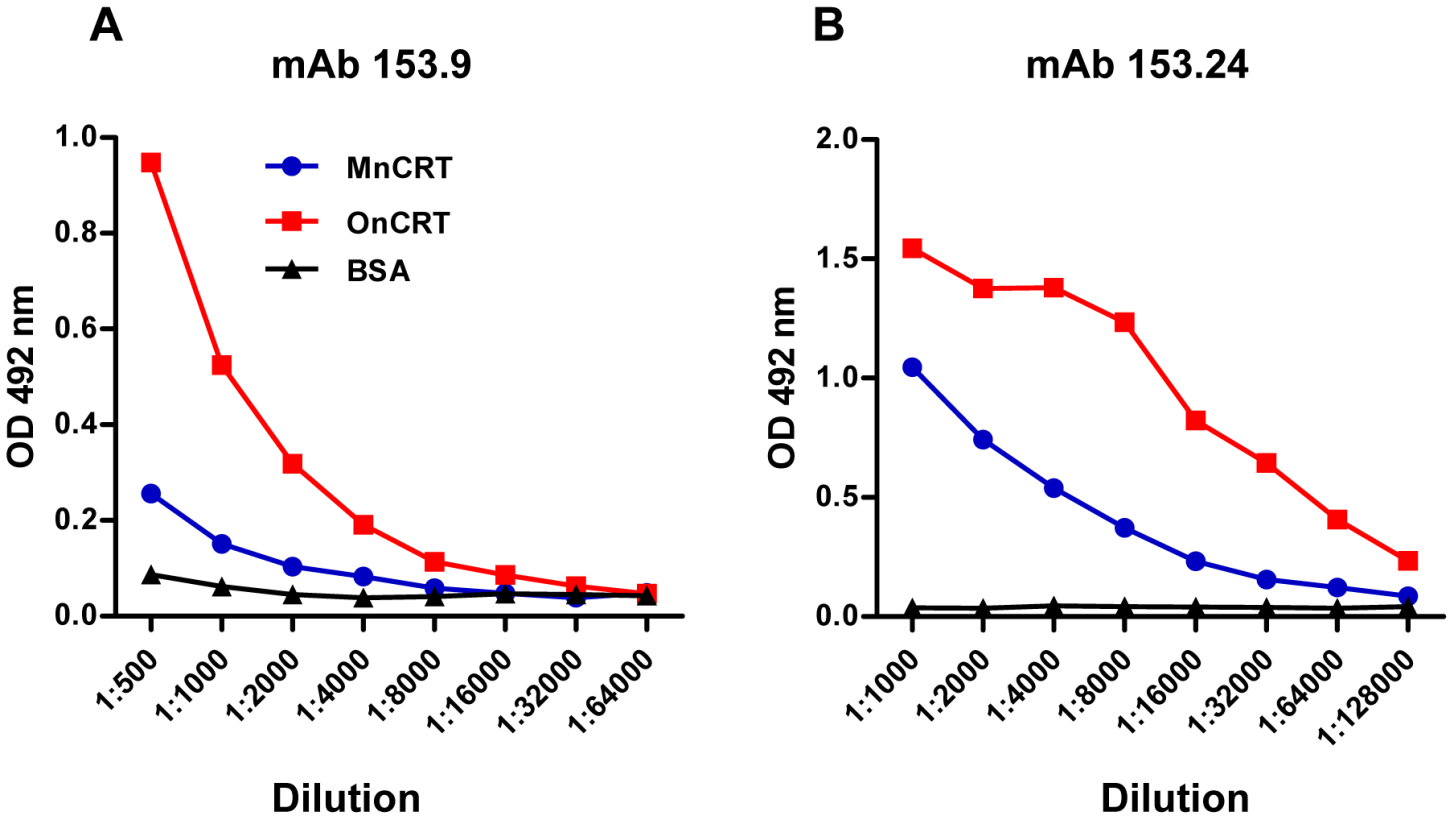
Differential recognition of MnCRT and OnCRT by anti-CRT mAbs. ELISA plates were pre-coated with MnCRT and OnCRT (2 ug/ml), serially diluted anti-CRT mAbs 153.9 (**A**) or 153.24 (**B**) were added in wells, followed by HRP-conjugated goat-anti-mouse IgG (1/2000 dilution) with OPD as substrate. Values are the mean OD492 nm± SD from triplicate wells. Results are representatives of 3 independent experiments.

As the molecules will form new or expose cryptic epitopes when they oligomerize [22], the recognition and binding ability with some mAbs will change, the oligomerization caused antigenicity alteration is detectable using certain mAbs with unique specificity. This phenomenon on nCRT was confirmed in the ELISA assay. Two CRT-specific mAbs (namely mAb 153.9 and mAb 153.24) were generated by immunizing mice with oligomeric rCRT [20]. As shown in Fig. 3, OnCRT was approximately 4 folds better recognized than MnCRT by these mAbs in ELISAs, indicating that some epitopes harbored by OnCRT were either absent or not easily accessible in MnCRT.

### Enhanced immunogenicity of OnCRT

**Figure 4:**
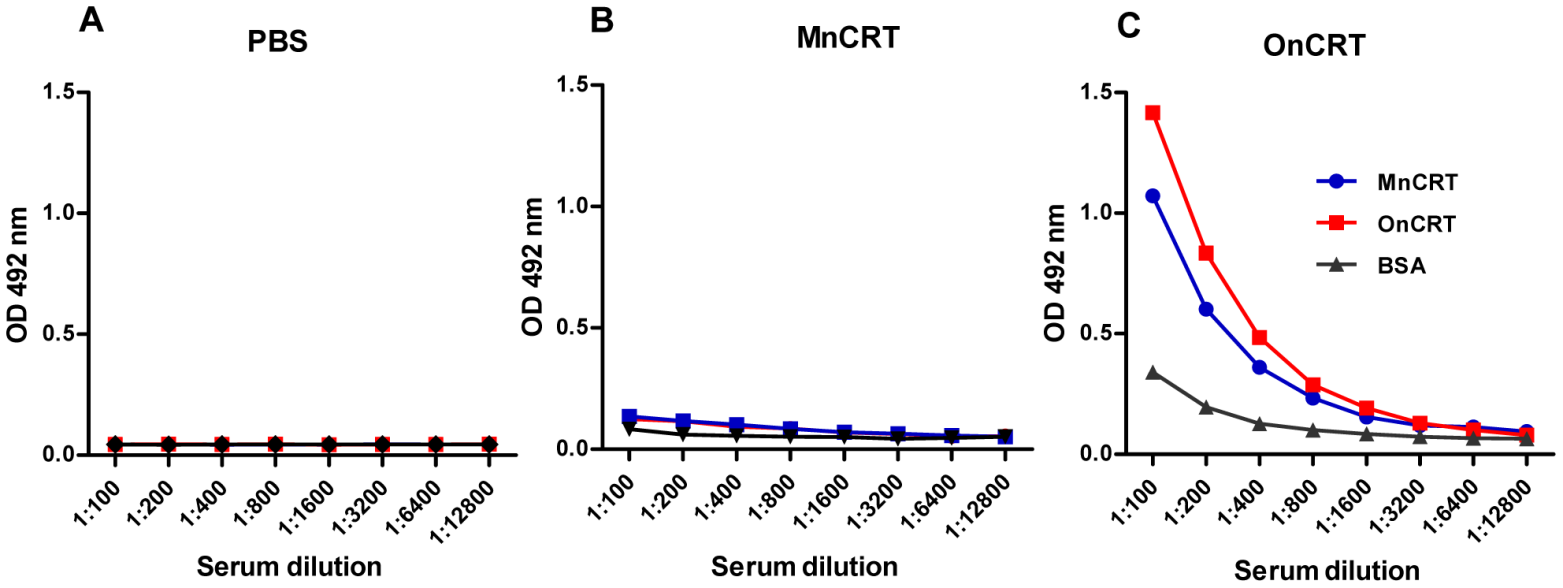
Induction of IgG responses by nCRT immunization in mice. BALB/c mice (5 per group) were s.c. immunized with PBS (**A**), MnCRT (**B**), or OnCRT (**C**) for 3 times. Serum samples, collected from these animals 9 days after the immunization, were assayed in ELISAs using plates that had been pre-coated with MnCRT, OnCRT, or BSA. The detection Abs were HRP-conjugated goat-anti-mouse IgG with OPD as substrate. Results are representatives of 3 independent experiments. Values are the mean OD492 nm± SD from triplicate cultures.

There is mounting evidence indicating that post-translational modification of proteins, including oligomerization, could lead to the generation of neoepitopes or exposure of cryptic epitopes that may subsequently trigger (auto)immune responses in vivo [29]–[32]. Compared with nCRT monomers, nCRT oligomers own possible novel epitopes, indicating that OnCRT may elicit stronger immune reaction in vivo. To elucidate this hypothesis, BALB/c mice were subcutaneously immunized with MnCRT or OnCRT (5 mice per group), and boosted twice with the same antigen preparations with 9 day intervals. Serum samples were collected from the immunized animals and analyzed using ELISAs for the presence of nCRT-specific Abs. As shown in Fig. 4, mice immunized with OnCRT, but not MnCRT or PBS, produced IgG Abs capable of recognizing OnCRT and, to a lesser extent, MnCRT, implying that tolerance of the mouse immune system towards nCRT was broken by immunization with OnCRT, but not with MnCRT. As expected, Abs thus produced preferentially recognized OnCRT, cross-recognition of MnCRT is likely the result of epitope spreading following tolerance break down in OnCRT-immunized mice. The CRT-specific IgG Abs did not seem to be immediately pathogenic, as no signs of inflammatory diseases in the mice up to six weeks after the immunization procedure.

**Figure 5:**
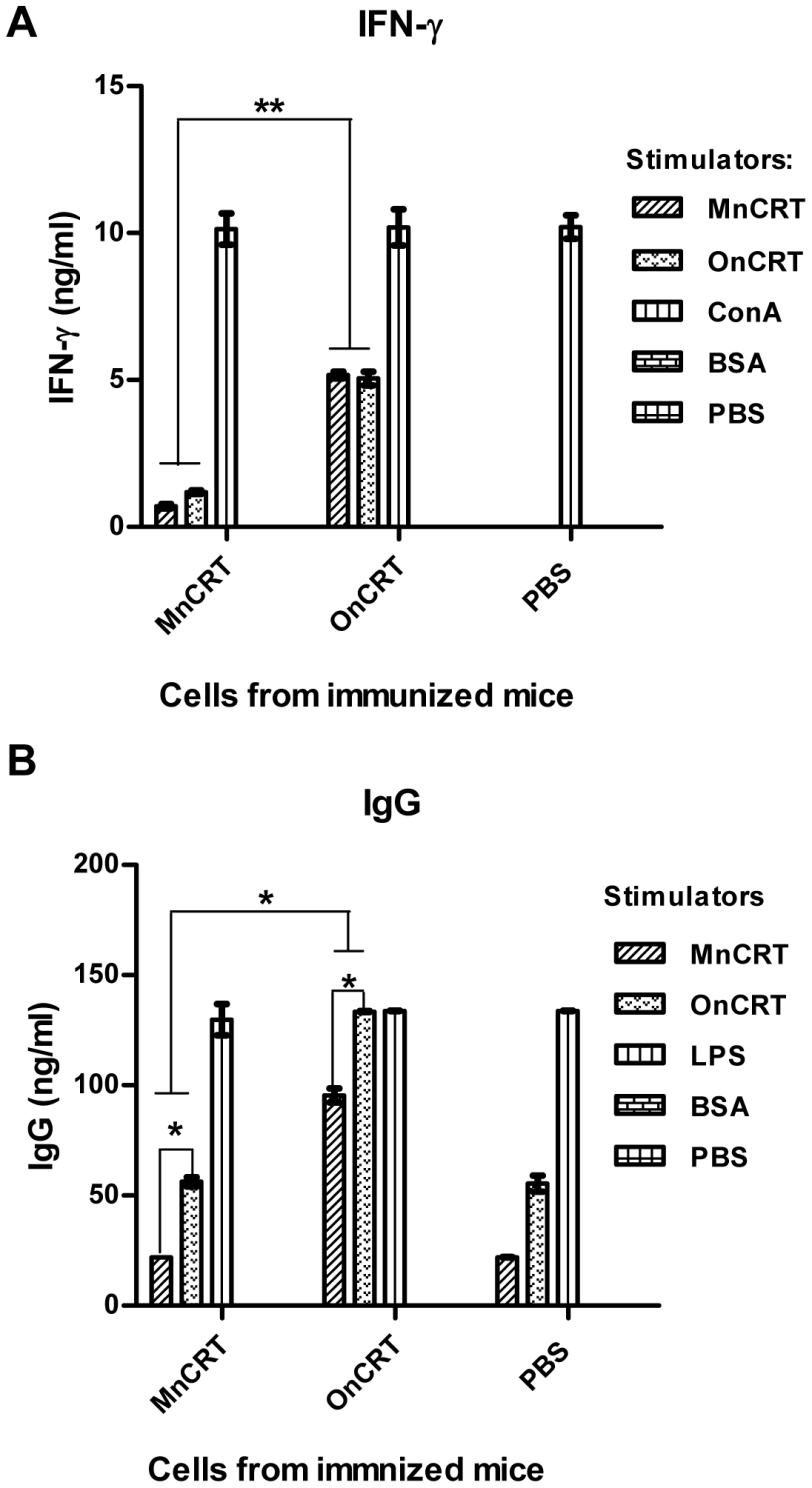
CRT-specific recall responses in vitro. Splenocytes (2.5×10^5^ cells/well) from mice that had been immunized as described in legend to Fig. 4 were stimulated with MnCRT or OnCRT for 6 days in vitro. Levels of IFN-γ (**A**) and total mouse IgG (**B**) in the culture supernatant were quantitated by ELISAs. PBS, BSA, ConA and LPS were included as negative and positive controls in the in vitro assays. Results are expressed as mean concentration (ng/ml) ± SD of triplicate wells and are representative of three independent experiments. *p<0.05.

Successful generation of Ag-specific IgG Abs in vivo is accompanied by Ag-specific activation and expansion of helper T (Th) and B lymphocytes. Indeed, splenocytes (containing both T and B lymphocytes) from the OnCRT-immunized mice responded significantly more vigorously than that from the control groups (immunized with MnCRT or PBS) to stimulation with nCRT in terms of IFN-γ and IgG production (Fig. 5).

### Enhanced stimulating effect of OnCRT on dendritic cells

**Figure 6:**
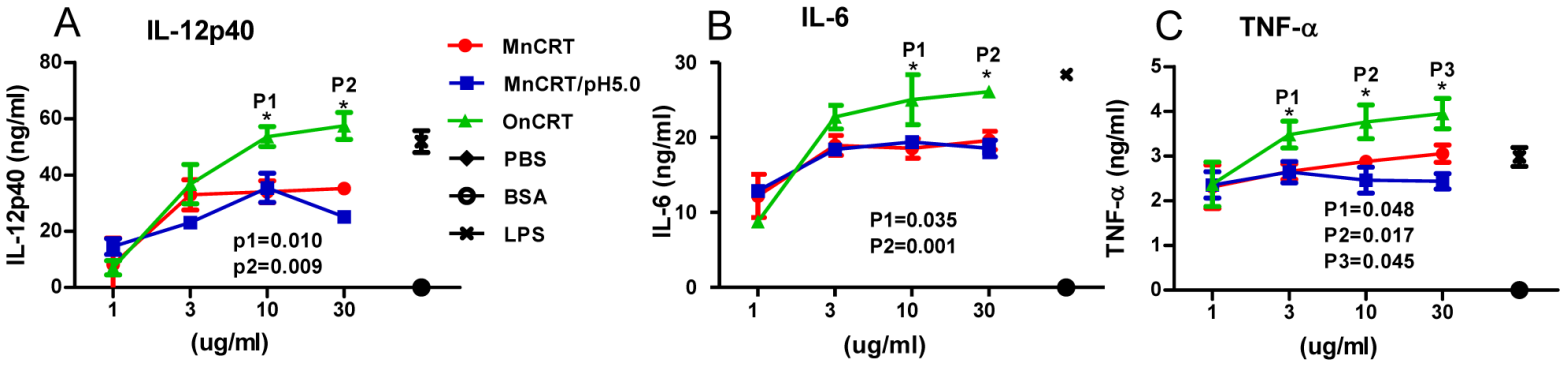
Augmented cytokine productions by BMDCs stimulated with OnCRT. BMDCs were cultured with different concentrations of MnCRT, MnCRT/pH 5.0, and OnCRT for 48 hrs, then IL-12p40(A), IL-6 (B), TNF-α(C) levels in the BMDCs culture supernatants were determined by ELISA. PBS, BSA (30 ug/ml) and LPS (300 ng/ml) were included as negative or positive controls, respectively. Results were expressed as mean concentration ± SD of triplicate cultures and are representative of three independent experiments. *p<0.05.

The different capabilities of OnCRT and MnCRT to elicit Ag-specific responses in vivo prompted us to further investigate their immunostimulatory potency against dendritic cells (DCs). To this end, we first generated BMDCs by culturing mouse bone marrow cells with GM-CSF and IL-4 for 7 days. The resultant BMDCs were then stimulated with MnCRT or OnCRT in 96-well plates and assayed for cytokine secretion in the culture supernatant. As shown in Fig. 6, although MnCRT were capable of inducing BMDCs to produce IL-12p40, IL-6 and TNF-α, significantly higher levels of these cytokines were detected in wells cultured with OnCRT.

## Discussion

In this study, we have illustrated for the first time that intracellular CRT as well as purified nCRT could oligomerize under “heat & low pH” conditions. More importantly, OnCRT appeared to be significantly more potent than MnCRT in terms of ability to break tolerance in mice (enhanced immunogenicity), generate cryptic epitope(s) recognizable by specific mAbs (altered antigenicity), induce IL-6, TNF-α, and IL-12p40 production by BMDCs in vitro (modulating antigen-presenting cells). These results are well in line with our previous observation that oligomeric rCRT fragments were extraordinarily potent as immunological stimulators [13], [20]. Given that homology between murine and human CRT is 94.2% and also that elevated levels of soluble CRT in body fluids correlate with the development of autoimmune disorders in humans [8], [20], the present results provide useful clues for our understanding on the potential immunopathological roles of circulating CRT in patients. However, direct evidence for the presence of circulating CRT oligomers either in patients with inflammatory autoimmune disorders or in animal models is not yet available. It would be of great interest to assess whether oligomeric CRT from patient sera or synovial fluid, if present, also exhibits similar immunological activities as OnCRT. Isolation of soluble CRT from patient blood turned out to be a formidable task, mainly due to the fact that CRT concentration in circulation is generally very low (less than 100 ng/ml in most cases). Another interesting point relating to the present work is whether anti-CRT autoantibodies in patients preferentially bind CRT oligomers over monomers. Development of sensitive assay kits using tools such as mAbs 153.24 and 153.9 will be valuable in attempts to address the question.

It should be emphasized that, in the “heat & low pH”-induced OnCRT preparations, only a relatively small percentage of the purified nCRT became oligomerized (Fig. 2B). More extreme treatment conditions led to formation of large insoluble aggregates rather than soluble oligomers (not shown). Given that only less than 1 mg nCRT was purified from 20 mouse livers, it was almost impossible to fractionate “pure” nCRT oligomers from OnCRT for additional experimentation. However, we argue that the “contaminating” MnCRT in our OnCRT preparations is unlikely to cause false positive results in our functional assays, as the MnCRT has been repeatedly shown to be non-immunogenic and much less active than oligomeric CRT. Moreover, we have previously reported that purified oligomeric rCRT fragments were at least 10 folds more active than rCRT monomers in terms of immune-stimulatory activities [13]. Unlike recombinant proteins expressed in prokaryotic systems, LPS contamination in our nCRT preparations from tissues, if any, is negligible: the endotoxin levels in 2 batches of nCRT preparations were below 1 pg/ml as determined by ELISA tests (data not shown), which is unlikely to have a noticeable effect in the in vitro and in vivo assays.

Cell stresses, including heat shock, exposure to heavy metals and perturbation of normal ER function, can up-regulate CRT expression [33], [34], which may in some cases lead to its secretion from cells. Mechanisms for CRT partial unfolding and possible oligomerization caused by chemical and physical (thermal) conditions have been studied in much detail [5], [18], [19], [35], [36]. Our BiFC experiments showed that intracellular CRT oligomerization could occur under 42°C at pH 6.5 (Fig. 1). Future experiments in this laboratory to assess the possibility that 293T cells co-expressing VN-CRT and VC-CRT fusion proteins can release (secret) oligomeric VN-CRT/VC-CRT following “heat & low pH” treatment. In any case, as body temperature can reach 42°C in patients with ultrahyperpyrexia and the microenvironment of inflamed tissues is acidic, it can be anticipated that oligomerization of soluble CRT may occur under certain pathological situations in vivo. Apart from “heat & low pH” condition, other situations may also result in oligomerization of CRT in vivo. For instance, Jorgensen et al have showed that the thermal behavior of CRT depends on pH and bundled Ca^2+^, loss of α-helical structure upon heating facilitates nCRT to oligomerize [5], [19]. Carpio et al recently demonstrated that arginylation also reduced CRT thermostability and induced a greater degree of oligomerization [35]. Arginylated CRT forms disulfide-bridged dimers that are increased in low Ca^2+^ conditions at physiological temperatures. Moreover, arginylated CRT self-oligomerization through noncovalent interactions is enhanced at temperatures above 40°C [35]. We also found that nCRT could oligomerize when maintained in the presence of EDTA at 42°C (data not shown). These results collectively support the possibility that CRT may oligomerize in vivo and contribute to the inflammatory immune reactions.

## Conclusions

Both extracellular and intracellular CRT can oligomerize under “heat & low pH” conditions, and oligomeric nCRT harbors novel epitopes, displays significantly increased abilities to stimulate DCs in vitro and elicit specific IgG responses in vivo. We conclude that under certain pathological situations soluble CRT in body fluid may exist in oligomeric form and play exacerbating roles in autoimmune diseases in patients.

## Supporting Information

Figure S1 **The antigen specificity of the mAbs 153.9 and 153.24.** ELISA plates were pre-coated with rCRT monomers and rCRT oligomers (2 ug/ml), serially diluted anti-CRT mAbs 153.9 (**A**) or 153.24 (**B**) were added in wells, followed by HRP-conjugated goat-anti-mouse IgG (1/2000 dilution) with OPD as substrate. Values are the mean OD492 nm ± SD from triplicate wells. Results are representatives of 3 independent experiments.
(TIF)

Checklist S1 **The ARRIVE checklist.**
(DOC)
