# Supplementary figures and images for: Immunological Activity Difference between Native Calreticulin Monomers and Oligomers
Source: PLoS One. 2014 Aug 29;9(8):e105502. doi: 10.1371/journal.pone.0105502 (PMC4149419; doi:10.1371/journal.pone.0105502)

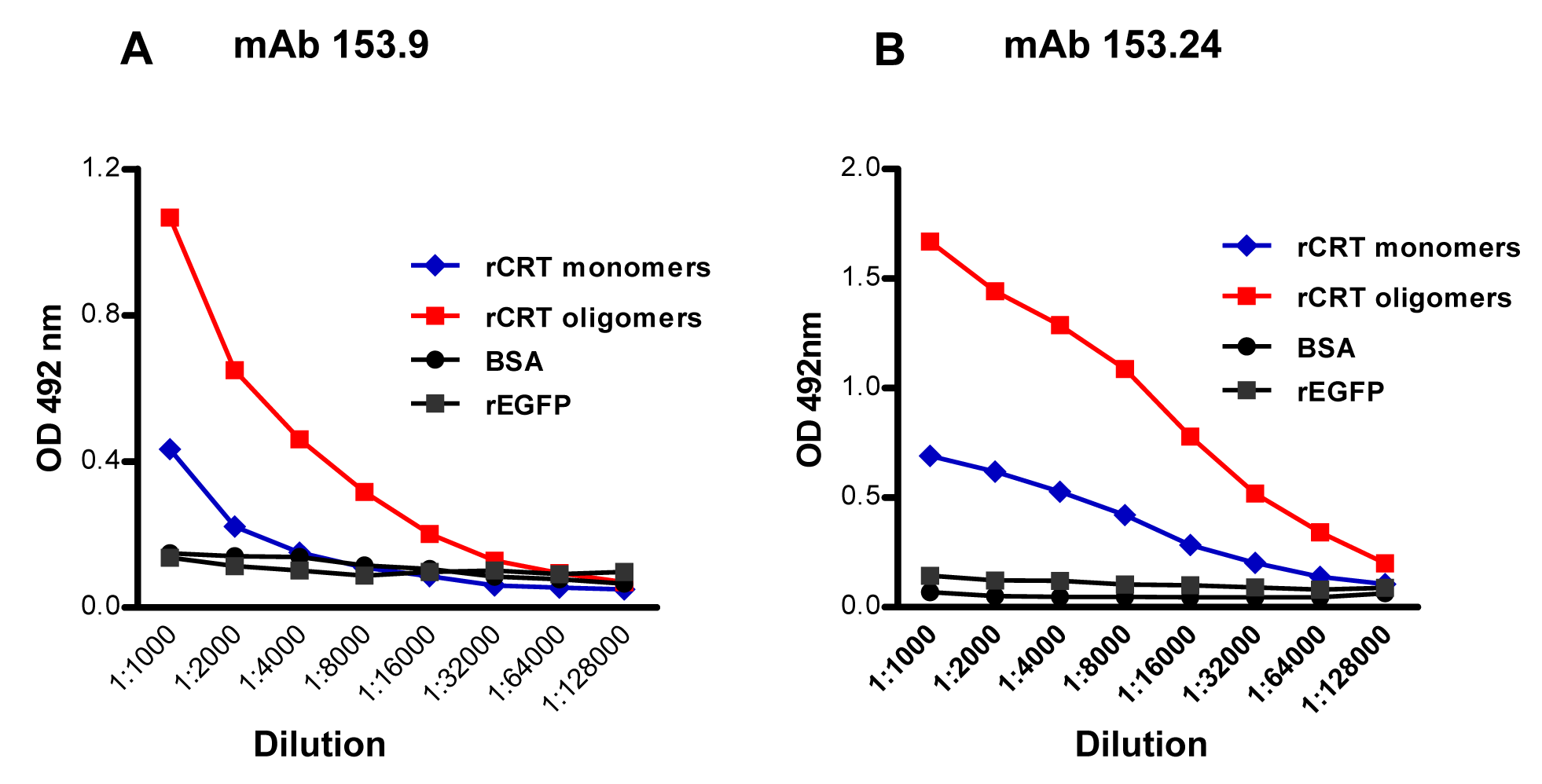

Supplement: Figure S1 — The antigen specificity of the mAbs 153.9 and 153.24. ELISA plates were pre-coated with rCRT monomers and rCRT oligomers (2 ug/ml), serially diluted anti-CRT mAbs 153.9 (A) or 153.24 (B) were added in wells, followed by HRP-conjugated goat-anti-mouse IgG (1/2000 dilution) with OPD as substrate. Values are the mean OD492 nm ± SD from triplicate wells. Results are representatives of 3 independent experiments. (TIF) [file pone.0105502.s001.tif]
